# Transmission of trisomy decreases with maternal age in mouse models of Down syndrome, mirroring a phenomenon in human Down syndrome mothers

**DOI:** 10.1186/s12863-016-0412-3

**Published:** 2016-07-11

**Authors:** Shani Stern, David Biron, Elisha Moses

**Affiliations:** Laboratory of Genetics, Salk Institute for Biological Studies, 10010 N. Torrey Pines Rd., La Jolla, CA 92037 USA; Department of Physics, James Franck Institute and the Institute for Biophysical Dynamics, University of Chicago, 929 E. 57th St GCIS E139F, Chicago, IL 60637 USA; Department of Physics of Complex Systems, Weizmann Institute of Science, P.O. Box 26, Rehovot, 76100 Israel

## Abstract

**Background:**

Down syndrome incidence in humans increases dramatically with maternal age. This is mainly the result of increased meiotic errors, but factors such as differences in abortion rate may play a role as well. Since the meiotic error rate increases almost exponentially after a certain age, its contribution to the overall incidence aneuploidy may mask the contribution of other processes.

**Results:**

To focus on such selection mechanisms we investigated transmission in trisomic females, using data from mouse models and from Down syndrome humans. In trisomic females the a-priori probability for trisomy is independent of meiotic errors and thus approximately constant in the early embryo. Despite this, the rate of transmission of the extra chromosome decreases with age in females of the Ts65Dn and, as we show, for the Tc1 mouse models for Down syndrome. Evaluating progeny of 73 Tc1 births and 112 Ts65Dn births from females aged 130 days to 250 days old showed that both models exhibit a 3-fold reduction of the probability to transmit the trisomy with increased maternal ageing. This is concurrent with a 2-fold reduction of litter size with maternal ageing. Furthermore, analysis of previously reported 30 births in Down syndrome women shows a similar tendency with an almost three fold reduction in the probability to have a Down syndrome child between a 20 and 30 years old Down syndrome woman.

**Conclusions:**

In the two types of mice models for Down syndrome that were used for this study, and in human Down syndrome, older females have significantly lower probability to transmit the trisomy to the offspring. Our findings, taken together with previous reports of decreased supportive environment of the older uterus, add support to the notion that an older uterus negatively selects the less fit trisomic embryos.

**Electronic supplementary material:**

The online version of this article (doi:10.1186/s12863-016-0412-3) contains supplementary material, which is available to authorized users.

## Background

Down Syndrome (DS) is the most abundant nonlethal chromosomal abnormality in humans, in which all or part of chromosome 21 appears in three copies instead of two. This gene imbalance results in cognitive impairment as well as in moderate to severe negative impacts on physical health [1, 2]. Since ~90 % of children with DS receive their extra chromosome from their mother [3, 4], the rates of DS and of other chromosomal abnormalities are known to increase with maternal age in humans [5, 6].

In humans, there is a reduction of survival of DS babies as compared to healthy ones primarily due to congenital heart disease [7–9]. In fact, most DS embryos will die in the uterus – previous studies report that the incidence of aneuploidy in spontaneous abortions is larger than 35 % [3, 10, 11]. They also reported that of the trisomies that can survive to birth, trisomy 21 was the most frequent one with a 2.3 % occurrence rate. However, in live births the occurrence rate of aneuploidy was only 0.3 %, of which trisomy 21 accounted for 0.13 %. Increase of the rate of aneuploidy with maternal age thus enhances the need for uterine selection (both positive and negative). The aging of the uterine may be playing a role in this selection process, as indicated by the approximately two-fold increase in the abortion rate with age [12–17].

It is particularly important to understand uterine selection because the ability to conceive is being extended to women over 50 years of age, with possible implication for IVF and genetic pre-screening [12–14]. At a high maternal age women are encouraged to monitor the health of the fetus through amniocentesis, despite the increase of risk for an abortion. This recommendation is linked to the reported exponential increase in the rate of chromosomal abnormalities with maternal age [10], but the possibility of higher selection against aneuploidy in older uteruses may affect the risk/benefit analysis of invasive screening procedures.

The origin of aneuploidy is still not fully understood, and several mechanisms play a role [18–20]. The relaxed selection hypothesis postulates that production of aneuploid gametes remains constant with maternal age, but that in utero selection against trisomic embryos decreases [21–25]. This hypothesis was challenged by findings indicating an increase in the likelihood of miscarriages of trisomic embryos with the age of healthy mothers [26–29]. Nevertheless, as the maternal age increases, the rate of aneuploidies in neonatal increases dramatically [30]. This results from the increment in the probability of meiotic errors with maternal age, which increases the rate of trisomic embryos. Thus, in healthy mothers, the dependence of negative selection against aneuploidies on age may be masked by meiotic errors. A model system in which the a priori probability for aneuploidy is fixed would enable to examine how selection is affected by maternal age. Observing progeny of mothers with aneuploidy (trisomy 21 in our case) fixes the a-priori probability of the aneuploidy at approximately the Mendelian ratio, i.e., at 50 %. We therefore use data from trisomic mothers, mice and human, to address this question.

While DS occurs in humans and not in rodents, several mouse models for DS have been developed for studying this disorder. In mice models for DS some of the genes orthologous to the genes of Homo sapiens (HSA) 21 are present at 3 copies. The Tc1 mouse model [31] contains a freely segregating human chromosome 21 with about 83 % of the known HSA 21 genes, and it is mosaic, with about 50 % of the cells containing the extra chromosome. The Ts65Dn mouse model [32] contains an extra translocation chromosome, containing genes from chromosome 16 and 17 of the mouse, and a total of ~65 % of DS genes in trisomy [33]. Both mouse models exhibit heart defects like human DS [34, 35]. Both mouse models were shown to perform poorly on various cognitive tasks as Morris water maze [31, 36]. Both have reduced long-term potentiation [31, 37]. The Ts65Dn mouse was shown to exhibit increase in inhibition [37, 38] and developmental delays, and both mouse models have been shown to have alterations in several ionic channel conductions [38].

In the Tc1 mouse model, the currently reported rate of transmission of the extra chromosome is approximately 40 % [31]. In the Ts65Dn mouse model an extra translocation chromosome is inherited. It was shown in [39] that while the extra chromosome in Ts65Dn is transmitted in the expected ratio of 50 % immediately after conception, there is a disproportionate loss of trisomic offspring in late gestation and after birth, similar to human DS [11]. It was also shown in [39] that as the Ts65Dn female ages, her litter size decreases and the transmission rate of the trisomy decrease concurrently.

We report here that in the Tc1 mouse model the ratio of trisomic to non-trisomic offspring diminishes with the age of the mother in a very similar distribution to Ts65Dn mothers. By extrapolation to embryonic stage, Tc1 females would also have a similar distribution of the Ts65Dn female, i.e. a 50 % (Mendelian) ratio for transmitting the extra (human) chromosome immediately after conception. Importantly, when we compare to previously reported cases of deliveries of women with DS, we find that a similar phenomenon appears and the probability of a child with DS diminishes with the age of the mother. The dependence of the fraction of trisomic progeny on the age of the mother is thus hypothesized to reflect the reduced fitness of trisomic embryos, who cannot survive the less supportive conditions of the older uterus. Our findings suggest that the observation of this phenomenon in trisomic mice is reproducible, mirrors the trend seen in human pregnancies, and can in the future be studied in these two independent mouse models.

## Methods

All procedures were approved by the Weizmann Institutional Animal Care and Use Committee.

### Grouping of sample sets

Genotyping was performed at one of three times. One group was genotyped at embryonic stage E17 (*n* = 28, 44 respectively for Tc1, Ts65Dn), another group immediately after birth at P0 (*n* = 10, 11 respectively for Tc1, Ts65Dn) and the rest after weaning (*n* = 35, 57 respectively for Tc1, Ts65Dn). In the latter group males were not always kept (*n* = 16, 35 respectively for Tc1, Ts65Dn), and these litters were not used for the analysis of litter size. During gestation females were typically weighed daily and their pregnancy was monitored. In two pregnancies of young Ts65Dn females, the entire progeny was lost after day 17. One young Tc1 and two young Ts65Dn mothers did not wean and the entire progeny was lost. In addition, two Tc1 and two Ts65Dn did not wean some of the pups, and consequently lost 10–20 % of their litter. In total these cases account for less than 6 % of the data and therefore could not have appreciably affected our results. For transmission of trisomy, analysis was performed on two sets of data: 1) litters where the entire progeny was genotyped 2) litters where we know the genotype of the females only (because the males were not kept, the ratio was therefore calculated as the number of trisomic females divided by the number of females in the litter), assuming that trisomy rate has a similar incidence in both male and female population. These two analyses were compared.

### Genotyping

Deoxyribonucleic acid (DNA) was extracted using the Extract-N-Amp Tissue polymerase chain reaction (PCR) kit from Sigma (http://www.sigmaaldrich.com/life-science/molecular-biology/dna-and-rna-purification/extract-n-amp.html), for each ~5 mg piece of brain or ~0.5 cm mouse tail. Genotyping of Ts65Dn was done according to the protocol described in [40]. For Tc1 mice we followed the protocol supplied courtesy of the Fisher lab, which is currently available in the Jackson homepage for genotyping of Tc1 [41]. An example of genotyping of Tc1 mice can be found in Additional file 1: Figure S1.

### Statistical analysis

The statistics for the human DS females was calculated by dividing the females into 2 age groups (below and above 25). The average age for the first group was 20, and for the second group it was 30. In each age group a score of ‘1’ was given to a DS baby, and a ‘0’ score to a healthy baby. The mean transmission rate for the trisomy 21 for each age group is then the mean of these scores. The standard deviation is similarly the standard deviation of these scores per each age group. The t-test was then performed for statistical significance using:

$$ t=\frac{\overline{x_1}-\overline{x_2}}{\sqrt{\left(\frac{\left({n}_1-1\right){s}_1^2+\left({n}_2-1\right){s}_2^2}{n_1+\left[{n}_2-2\right]}\right)\left(\frac{1}{n_1}+\frac{1}{n_2}\right)}} $$

And a *p* value evaluated using *n*_1_ + *n*_2_ – 2 degrees of freedom.

In the mice population, the females were divided into 2 age groups: below and above 200 days. The average maternal age for the first groups was approximately 130 days and for the second it was 250 days. The transmission probability of each progeny was calculated as the ratio of the number of trisomic pups and the litter size. The statistics for the mice population was performed using a two-sample t-test on the two sets (young and old mothers) of transmission of trisomy probabilities. Rejection of the null hypothesis indicates that the means of the two sets are different from each other. Since the older females have lower fertility, some of the graphs presented were obtained with small n’s. [42, 43] have shown that the t-test holds with a good approximation for n’s as low as four. Similarly we performed a t-test between the two maternal age groups on the litter size.

## Results

### The probability of trisomic pups in trisomic Tc1 DS model females decreases with age

**Fig. 1:**
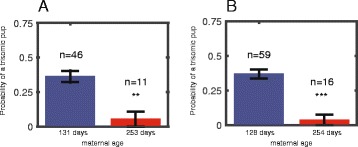
Transmission of trisomy reduces with maternal age in Tc1 model mice. **a** Probability of a trisomic pup for females younger than 200 days (*n* = 46) is ~ 10 fold higher than for females older than 200 days (*n* = 11, *p* = 0.0011). The analysis was performed only for litters where the genotype for the entire progeny is known. **b** Probability of a trisomic pup for females younger than 200 days (*n* = 59) is ~ 10 fold higher than for females older than 200 days (*n* = 16, *p* = 0.000055). The analysis was performed for the entire dataset. For some of the dataset only female information exists (see methods)

We genotyped progeny of 75 Tc1 DS model females and analyzed the rate of the trisomy with respect to the female’s age. Maternal age was grouped in one of two groups: below or above 200. In a subset of the data (57 out of the 75 progenies), where the genotypes of all male and female pups were identified, the average probabilities for having a trisomic pup in the two groups were 0.36 ± 0.04 (*n* = 46) for younger females and 0.05 ± 0.05 (*n* = 11) for older females (Total 57 = 46 + 11). The mean maternal age of the two groups was 131 and 253 days (Fig. 1a). Thus, the probability to transmit the extra chromosome was significantly lower in the group of older trisomic mothers (*p* = 0.0011). When analyzing the entire data set (including the cases in which the genotypes of the male pups were not assayed, total of 75, see methods), the rates of transmission of trisomy were similar: 0.37 ± 0.03 (*n* = 59) and 0.04 ± 0.04 (*n* = 16) for the younger and older trisomic mother groups, respectively (*p* = 0.00055) (Total 75 = 59 + 16). The mean maternal age was 128 days and 254 days (Fig. 1b).

### Litter size of trisomic Tc1 DS model females decreases with age

**Fig. 2:**
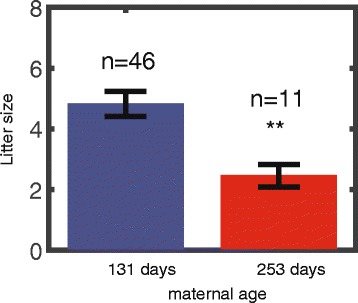
Litter size decreases with maternal age in DS model mice. Litter size of females younger than 200 days (*n* = 46) is ~ 2 fold higher than for females older than 200 days (*n* = 11, *p* = 0.0081). The analysis was performed only for litters where the genotype for the entire progeny is known

We genotyped progeny of 57 Tc1 DS model females and analyzed the litter size as a function of maternal age (the subset of the data out of the entire 75 for which we had the genotyping of both the male and female pups). Maternal age was grouped in one of two groups: Below or above 200 days with average ages of 131 and 253 days. The average litter size for the younger and older maternal age groups was 4.8 ± 0.4 (*n* = 46) and 2.5 ± 0.4 (*n* = 11), respectively (Fig. 2). Thus, the litter size of the older trisomic mothers age group was significantly smaller than that of the younger group (*p* = 0.008).

### The probability to have trisomic pups in trisomic Ts65Dn DS model females decreases with age

**Fig. 3:**
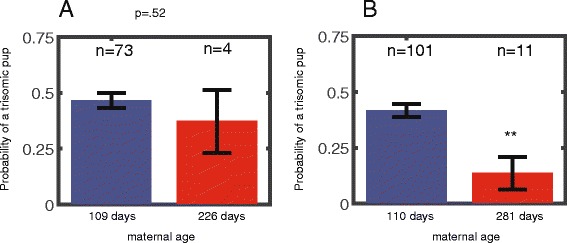
Transmission of trisomy reduces with maternal age in Ts65Dn model mice. **a** Probability of a trisomic pup for females younger than 200 days (*n* = 73) and for females older than 200 days (*n* = 4, *p* = 0.52). The analysis was performed only for litters where the genotype for the entire progeny is known. **b** Probability of a trisomic pup for females younger than 200 days (*n* = 101) is ~ 3 fold higher than for females older than 200 days (*n* = 11, *p* = 0.0029). The analysis was performed for the entire dataset. For some of the dataset only female information exists (see Methods)

We genotyped progeny of 112 Ts65Dn DS model females and analyzed the rate of the trisomy with respect to the female’s age. Maternal age was grouped in one of two groups: below or above 200. In a subset of the data (77 out of the 112 progenies), where the genotypes of all male and female pups were identified, the average probabilities for having a trisomic pup in two groups were 0.46 ± 0.03 (*n* = 73) for younger females and 0.37 ± 0.14 (*n* = 4) for older females (*p* = 0.52) (Fig. 3a) (Total 77 = 73 + 4). The average maternal age was 109 days for the younger group and 226 days for the older females. When analyzing the entire data set (including the cases in which the genotypes of the male pups was not assayed, total of 112, see methods), the rates of transmission of trisomy probability for the younger trisomic mother were 0.42 ± 0.03 (*n* = 101) for the younger females and 0.14 ± 0.07 (*n* = 11) for the older females, *p* = 0.0029 (Total 112 = 101 + 11). The average maternal age was 110 days and 281 days in the two groups (Fig. 3b).

### Litter size in trisomic Ts65Dn DS model females decrease with age

**Fig. 4:**
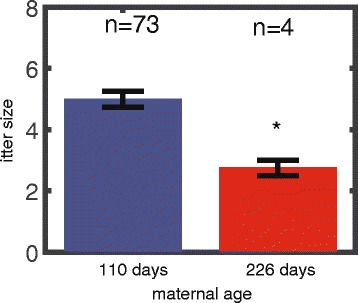
Litter size decreases with maternal age in DS model mice. Litter size of females younger than 200 days (*n* = 73) is ~ 2 fold higher than for females older than 200 days (*n* = 4, *p* = 0.0213). The analysis was performed only for litters where the genotype for the entire progeny is known

We genotyped progeny of 77 Ts65Dn DS model females and analyzed the litter size as a function of maternal age (the subset of the data out of the entire 112, for which we had the genotyping of both the male and female pups). Maternal age was grouped in one of two age groups: below or above 200 days with average ages of 109 and 226 days. The average litter sizes for the younger and older maternal age groups were 5 ± 0.3 (*n* = 73) and 2.8 ± 0.3 (*n* = 4), respectively (Fig. 4). Thus, the older age group of trisomic mothers exhibited significantly smaller litter sizes (*p* = 0.0213).

### Women with DS become less likely to deliver a DS child with increased age

We calculated the rate of DS (trisomy 21) babies as a function of maternal age in *n* = 30 reported cases of DS mother deliveries [44–50].

**Fig. 5:**
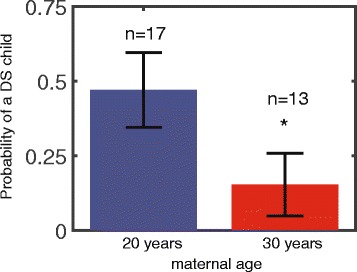
Analysis of previously reported cases of Down syndrome women reveals a lower probability for a baby with DS as the mother ages. *N* = 30 cases of Down syndrome mothers were analyzed in two age groups: above and below 25 years of age. For DS mothers with an average age of 20 the probability for having a DS child is approximately 3 times higher than for women with an average age of 30 (*p* = 0.042)

The dataset we analyzed included DS mothers of ages 17–35 years. We divided these cases to two age groups: less than 25 and 25 and above. Figure 5 shows the frequency of trisomy 21 babies in each of the age groups (with average maternal ages of 20 and 30 years). The transmission rates in the younger and older age groups were 0.47 ± 0.12 (*n* = 17) and 0.17 ± 0.11 (*n* = 13), respectively, *p* value = 0.042. Our analysis suggests that the probability of delivering a DS trisomic baby by a DS mother decreases with maternal age by approximately 3 fold.

### Mouse vs. human mothers

**Fig. 6:**
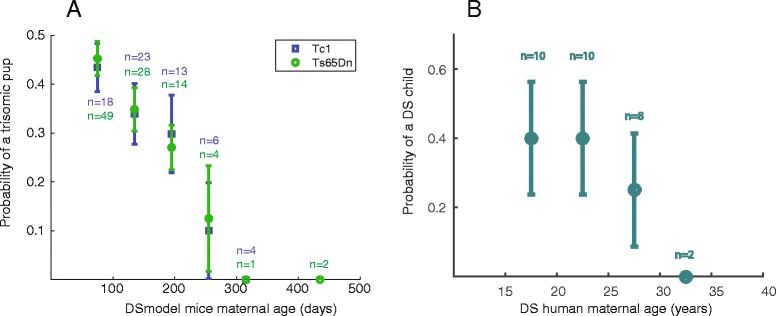
A gradual decrease in probability to transmit trisomy to offspring with maternal age is seen both in DS model mice and in DS women. **a** Maternal age in DS model mice was grouped into bins of 45 days. A gradual and similar decrease in the probability to transmit the trisomy with maternal age is seen for both Tc1 and Ts65Dn model mice. **b** Maternal age in human DS was grouped into 4 bins of 5 years groups. Similar to the mice, a gradual decrease in the probability to transmit trisomy with maternal age is evident

Figure 6 compares data from human DS mothers and mouse DS models, divided to 4 and 7 age groups, respectively. Figure 6a shows that the trisomy transmission rate falls similarly for both DS mice models from 0.43 ± 0.06 in 75 days old females to ~0 for females older than 300 days. Figure 6b shows that the data from human DS mothers mirrors this trend. The frequency of trisomy 21 babies as a function of maternal age falls from 0.4 ± 0.19 in the 17.5 years age group to 0 in the 32.5 years age group. We therefore suggest the two DS model mice as a model organism in which the trend of in-uterus selection against trisomy 21 embryos can be studied. This presents a new toolset with which this question can be addressed.

## Discussion

Both Ts65Dn and Tc1 DS mouse models have reduced fertility when compared to WT mice. The rate of conceptions to mating reduces drastically with maternal age. We approximate the rate to be ~1:3 in younger females (~3 months old) and as the female ages the rate reduces to approximately 1:10 (in a female ~8 months old). The litter size is smaller than the WT litter size and as shown here also reduces with maternal age. We show that older females with DS exhibit a significantly reduced chance of giving birth to offspring with DS.

Litter size in mice is known to decrease with maternal age. For example, in [51, 52] it was reported that in Bj44Gy mice a young female has an average litter size of ~7 pups, while a 9–10 months old female has an average litter size of less than 3. One possible cause for the decrease in litter size is an increased rate of mutations with increasing maternal age, which enhances the chances of producing unviable embryos. Another possibility is that the uterus of older females provides a less supportive environment. The answer provided in [51] leans towards the second possibility, showing that although the anomalous embryo rate does go up from 4.8 % in young females (2–7 months old) to 12.1 % in old ones (8–12 months old), this still does not explain the drastic reduction in litter size. Finn [13] also supports the reduction in progeny size to be related to lack of supportive environment in the old uterus. Transplantation of embryos into young or old hosts was used [52] to show that only 14 % of transplantation in old females survived to term while 48 % survived in young females, suggesting that older females do indeed have a less favorable uterine environment.

These results point at an interesting balance of competing pressures that together determine the ratio of DS births in healthy human females as they age. On one hand the number of aneuploidies in the gamete rises with age [5, 11, 53]. On the other hand, healthy gametes and embryos are strongly selected [10, 11]. Less favorable conditions in the older uterus may negatively impact the survival of less fit embryos, but this was not directly demonstrated before. Observing the progeny of DS women and of DS model female mice allows the isolation of the negative selection of the older uterus on the survival of DS babies or trisomic pups. When the female is healthy there is an a-priori higher chance for trisomy as the female ages due to meiotic errors, but the use of DS trisomic females (both mice and women) gives a fixed a-priory chance of ~50 % for a trisomic embryo irrespective of age. Thus the rate of trisomy may predominantly depend on the survival of these embryos in the uterus.

In addition to uterine selection, one should consider the possibility that the increase in rate of chromosomal abnormalities with maternal age may preferentially impact the survival of trisomic embryos. However, the change in spontaneous rate for anomalous mouse embryo for older females is less than 10 % [53]. In humans we can estimate an upper bound for the possible effect by noting that the rate of aneuploidy is reported to increase from about 3–15 % in young mothers to about 30 % in older ones [10, 54, 55]. Of these genetic anomalies more than 90 % are lethal even on a normal genetic background [54, 56]. To the best of our knowledge, these additional abnormalities are statistically independent of the inherited genetic background of the embryo. Thus, when considering how the fraction of live DS offspring changes with the age of a DS mother, even under the strictest of assumptions the maximal effect of an additional aneuploidy is minuscule (about 20 fold smaller than the three fold change we observe). Indeed, the design of our experiment ensures that the dominant genetic pathology in the offspring is the DS one. These considerations lead us to favor the possibility of uterine selection against DS offspring over an effect of additional aneuploidy. Our study, however, does not identify the time point of gestation at which the DS offspring are lost.

Collectively, previous findings and ours demonstrate that in rodents and in humans age is negatively correlated with the survival of trisomic progeny. In Tc1 and Ts65Dn DS model mice females, both the litter size and the probability to produce trisomic pups decrease with age. Interestingly, E9.5 Ts65Dn embryos exhibit the expected Mendelian ratio of DS positive embryos [20], but older DS positive embryos and pups are lost at a higher rate than euploid ones. The similar distributions we show for the rate of trisomy as a function of maternal age (Fig. 6a), suggest that the extrapolation to embryonic phase would give a similar distribution for Tc1 females as was shown in [39] for Ts65Dn females, i.e. a Mendelian distribution for inheritance of the extra chromosome. Comparing to Fig. 6b for women suggests that these rates can be used to model the corresponding rates in a human population. In particular, DS women likely have an approximately Mendelian distribution of transmitting the extra human 21 chromosome to their babies. The similarities between the mice and human distributions offer a tool for measurement of selective pressure against trisomic progeny.

## Conclusions

We have shown that in two completely genetically different Down syndrome mouse models, the trisomic female has a significantly reduced probability of having viable Down syndrome pups with increasing age. An analysis of reported case studies of Down syndrome human mothers shows similarly a significantly reduced probability of delivering a baby with Down syndrome as the mother ages. These results are a strong indication of increased *in-utero* selection against Down syndrome offspring with increased maternal age. The study of trisomic mothers allows observation of this selection mechanism against the trisomic offspring, since for diploid females, it is masked by the large increase in meiotic errors with maternal age.

## Abbreviations

DNA, deoxyribonucleic acid; DS, down syndrome; HSA, homo sapiens; PCR, polymerase chain reaction

## Additional file

Additional file 1: Figure S1. Genotyping Tc1. An example picture of a gel used during genotyping. Two lines refer to a Tc1 positive trisomic pup. One line refers to a disomic pup. (EPS 1781 kb)

## References

[1] Smith DS (2001). Health care management of adults with Down syndrome. Am Fam Physician.

[2] van Allen MI, Fung J, Jurenka SB (1999). Health care concerns and guidelines for adults with Down syndrome. Am J Med Genet.

[3] Hassold T, Hunt P (2001). To err (meiotically) is human: the genesis of human aneuploidy. Nat Rev Genet.

[4] Serra A, Neri G (1990). Trisomy 21: conference report and 1990 update. Am J Med Genet Suppl.

[5] Gaulden ME (1992). Maternal age effect: the enigma of Down syndrome and other trisomic conditions. Mutat Res.

[6] Nicolaides KH (2004). Nuchal translucency and other first-trimester sonographic markers of chromosomal abnormalities. Am J Obstet Gynecol.

[7] Frid C, Drott P, Otterblad Olausson P, Sundelin C, Anneren G (2004). Maternal and neonatal factors and mortality in children with Down syndrome born in 1973–1980 and 1995–1998. Acta Paediatr.

[8] Halliday JL, Watson LF, Lumley J, Danks DM, Sheffield LJ (1995). New estimates of Down syndrome risks at chorionic villus sampling, amniocentesis, and livebirth in women of advanced maternal age from a uniquely defined population. Prenat Diagn.

[9] Morris JK, Wald NJ, Watt HC (1999). Fetal loss in Down syndrome pregnancies. Prenat Diagn.

[10] Hassold T, Abruzzo M, Adkins K, Griffin D, Merrill M, Millie E, Saker D, Shen J, Zaragoza M (1996). Human aneuploidy: incidence, origin, and etiology. Environ Mol Mutagen.

[11] Nagaoka SI, Hassold TJ, Hunt PA (2012). Human aneuploidy: mechanisms and new insights into an age-old problem. Nat Rev Genet.

[12] Kong S, Zhang S, Chen Y, Wang W, Wang B, Chen Q, Duan E, Wang H (2012). Determinants of uterine aging: lessons from rodent models. Sci China Life Sci.

[13] Finn CA (1962). Embryonic death in aged mice. Nature.

[14] Brosens JJ, Salker MS, Teklenburg G, Nautiyal J, Salter S, Lucas ES, Steel JH, Christian M, Chan YW, Boomsma CM (2014). Uterine selection of human embryos at implantation. Sci Rep.

[15] Macklon NS, Brosens JJ (2014). The human endometrium as a sensor of embryo quality. Biol Reprod.

[16] Nybo Andersen AM, Wohlfahrt J, Christens P, Olsen J, Melbye M (2000). Maternal age and fetal loss: population based register linkage study. BMJ.

[17] Menken J, Trussell J, Larsen U (1986). Age and infertility. Science.

[18] Allen EG, Freeman SB, Druschel C, Hobbs CA, O’Leary LA, Romitti PA, Royle MH, Torfs CP, Sherman SL (2009). Maternal age and risk for trisomy 21 assessed by the origin of chromosome nondisjunction: a report from the Atlanta and National Down Syndrome Projects. Hum Genet.

[19] Petersen MB, Mikkelsen M (2000). Nondisjunction in trisomy 21: origin and mechanisms. Cytogenet Cell Genet.

[20] Rowsey R, Kashevarova A, Murdoch B, Dickenson C, Woodruff T, Cheng E, Hunt P, Hassold T (2013). Germline mosaicism does not explain the maternal age effect on trisomy. Am J Med Genet A.

[21] Ayme S, Lippman-Hand A (1982). Maternal-age effect in aneuploidy: does altered embryonic selection play a role?. Am J Hum Genet.

[22] Kline J, Stein Z, Susser M, Warburton D (1986). Induced abortion and the chromosomal characteristics of subsequent miscarriages (spontaneous abortions). Am J Epidemiol.

[23] Stein Z, Susser M, Warburton D, Wittes J, Kline J (1975). Spontaneous abortion as a screening device. The effect of fetal survival on the incidence of birth defects. Am J Epidemiol.

[24] Drugan A, Yaron Y, Zamir R, Ebrahim SA, Johnson MP, Evans MI (1999). Differential effect of advanced maternal age on prenatal diagnosis of trisomies 13, 18 and 21. Fetal Diagn Ther.

[25] Neuhauser M, Krackow S (2007). Adaptive-filtering of trisomy 21: risk of Down syndrome depends on family size and age of previous child. Naturwissenschaften.

[26] Spandorfer SD, Davis OK, Barmat LI, Chung PH, Rosenwaks Z (2004). Relationship between maternal age and aneuploidy in in vitro fertilization pregnancy loss. Fertil Steril.

[27] Warburton D (1989). The effect of maternal age on the frequency of trisomy: change in meiosis or in utero selection?. Prog Clin Biol Res.

[28] Hook EB (1983). Down syndrome rates and relaxed selection at older maternal ages. Am J Hum Genet.

[29] Fragouli E, Wells D, Whalley KM, Mills JA, Faed MJ, Delhanty JD (2006). Increased susceptibility to maternal aneuploidy demonstrated by comparative genomic hybridization analysis of human MII oocytes and first polar bodies. Cytogenet Genome Res.

[30] Chiang T, Schultz RM, Lampson MA (2012). Meiotic origins of maternal age-related aneuploidy. Biol Reprod.

[31] O’Doherty A, Ruf S, Mulligan C, Hildreth V, Errington ML, Cooke S, Sesay A, Modino S, Vanes L, Hernandez D (2005). An aneuploid mouse strain carrying human chromosome 21 with Down syndrome phenotypes. Science.

[32] Davisson MT, Schmidt C, Reeves RH, Irving NG, Akeson EC, Harris BS, Bronson RT. Segmental trisomy as a mouse model for Down syndrome. Prog Clin Biol Res. 1993;384:117–33. 8115398

[33] Akeson EC, Lambert JP, Narayanswami S, Gardiner K, Bechtel LJ, Davisson MT (2001). Ts65Dn -- localization of the translocation breakpoint and trisomic gene content in a mouse model for Down syndrome. Cytogenet Cell Genet.

[34] Dunlevy L, Bennett M, Slender A, Lana-Elola E, Tybulewicz VL, Fisher EM, Mohun T (2010). Down’s syndrome-like cardiac developmental defects in embryos of the transchromosomic Tc1 mouse. Cardiovasc Res.

[35] Williams AD, Mjaatvedt CH, Moore CS (2008). Characterization of the cardiac phenotype in neonatal Ts65Dn mice. Dev Dyn.

[36] Reeves RH, Irving NG, Moran TH, Wohn A, Kitt C, Sisodia SS, Schmidt C, Bronson RT, Davisson MT (1995). A mouse model for Down syndrome exhibits learning and behaviour deficits. Nat Genet.

[37] Kleschevnikov AM, Belichenko PV, Villar AJ, Epstein CJ, Malenka RC, Mobley WC (2004). Hippocampal long-term potentiation suppressed by increased inhibition in the Ts65Dn mouse, a genetic model of Down syndrome. J Neurosci.

[38] Stern S, Segal M, Moses E (2015). Involvement of Potassium and Cation Channels in Hippocampal Abnormalities of Embryonic Ts65Dn and Tc1 Trisomic Mice. EBioMedicine.

[39] Roper RJ, St John HK, Philip J, Lawler A, Reeves RH (2006). Perinatal loss of Ts65Dn Down syndrome mice. Genetics.

[40] Reinholdt LG, Ding Y, Gilbert GJ, Czechanski A, Solzak JP, Roper RJ, Johnson MT, Donahue LR, Lutz C, Davisson MT (2011). Molecular characterization of the translocation breakpoints in the Down syndrome mouse model Ts65Dn. Mamm Genome.

[41] Jackson (2010). Protocol for genotyping TC1 mice.

[42] Student (1908). The Probable Error of a Mean. Biometrika.

[43] Winter JCF. Using the Student’s t-test with extremely small sample sizes. Practical Assessment, Research and Evaluation. 2013;18(10):1-12.

[44] Bovicelli L, Orsini LF, Rizzo N, Montacuti V, Bacchetta M (1982). Reproduction in Down syndrome. Obstet Gynecol.

[45] Kaushal MBA, Kadi P, Karandae J, Baxi D (2010). Woman with Down Syndrome delivered a Normal Child. Int J Infertil Fetal Med.

[46] Kristesashvili DI (1988). Offspring of patients with Down syndrome. Genetika.

[47] Liu XY, Jiang YT, Wang RX, Luo LL, Liu YH, Liu RZ (2015). Inheritance of balanced translocation t(17; 22) from a Down syndrome mother to a phenotypically normal daughter. Genet Mol Res.

[48] Santo LMAMLDE (2013). Marriage and reproduction in a woman with Down syndrome. International Medical Review on Down’s Syndrome.

[49] Scharrer S, Stengel-Rutkowski S, Rodewald-Rudescu A, Erdlen E, Zang KD (1975). Reproduction in a female patient with Down’s syndrome. Case report of a 46, XY child showing slight phenotypical anomalies, born to a 47, XX, + 21 mother. Humangenetik.

[50] Shobha Rani A, Jyothi A, Reddy PP, Reddy OS (1990). Reproduction in Down’s syndrome. Int J Gynaecol Obstet.

[51] Gosden RG (1973). Chromosomal anomalies of preimplantation mouse embryos in relation to maternal age. J Reproduction Fertility.

[52] Talbert GB, Krohn PL (1966). Effect of maternal age on viability of ova and uterine support of pregnancy in mice. J Reprod Fertil.

[53] Dailey T, Dale B, Cohen J, Munne S (1996). Association between nondisjunction and maternal age in meiosis-II human oocytes. Am J Hum Genet.

[54] Bahce M, Cohen J, Munne S (1999). Preimplantation genetic diagnosis of aneuploidy: were we looking at the wrong chromosomes?. J Assist Reprod Genet.

[55] Rabinowitz M, Ryan A, Gemelos G, Hill M, Baner J, Cinnioglu C, Banjevic M, Potter D, Petrov DA, Demko Z (2012). Origins and rates of aneuploidy in human blastomeres. Fertil Steril.

[56] Munne S, Bahce M, Sandalinas M, Escudero T, Marquez C, Velilla E, Colls P, Oter M, Alikani M, Cohen J. Differences in chromosome susceptibility to aneuploidy and survival to first trimester. Reproductive biomedicine online. 2004; 8(1):81-90. 10.1016/s1472-6483(10)60501-9 14759293

